# Comparison of Electron Compton Scattering with Positron Compton Scattering in Polyethylene

**DOI:** 10.3390/ma18071609

**Published:** 2025-04-02

**Authors:** Maurizio Dapor

**Affiliations:** 1 European Centre for Theoretical Studies in Nuclear Physics and Related Areas, Fondazione Bruno Kessler, 38123 Trento, Italy; dapor@ectstar.eu; 2 Trento Institute for Fundamental Physics and Applications (TIFPA-INFN), 38123 Trento, Italy

**Keywords:** elastic peak electron spectroscopy, electron Compton scattering, positron Compton scattering, Monte Carlo simulation

## Abstract

Understanding the interaction of charged particles with polymers is crucial for applications in materials science, radiation physics, and electron spectroscopy. This study investigates the differences in the elastic scattering spectra of electrons and positrons in polyethylene, focusing on the underlying mechanisms that influence the spectral features. The analysis isolates key factors such as recoil energy, Doppler broadening, and the interplay between elastic and inelastic mean free paths. Using Monte Carlo simulations, we analyze the effects of the elastic and inelastic mean free paths on the intensity of the elastic peaks in an energy range from 1000 eV to 3000 eV. The results show that the elastic peaks are consistently more intense for electrons than for positrons, correlating with the differences in the respective elastic scattering cross sections. In addition, we evaluate the effects of different inelastic mean free path models on spectral variations and compare the simulated data showing how variations in inelastic mean free path values affect the intensity of elastic peaks and the elastic reflection coefficient of polyethylene. The percentage difference in the elastic reflection coefficients of electrons and positrons in polyethylene decreases from 49% to 24% when the incident particle energy increases from 1000 eV to 3000 eV. These findings contribute to a refined understanding of the interactions of electrons and positrons with polymers, improve the accuracy of Monte Carlo simulations, and promote methods for material characterization.

## 1. Introduction

The study of the interactions of electrons and positrons with matter is of fundamental importance in many scientific and technological fields, e.g., surface research, the analysis of radiation damage, and the characterization of polymers. In particular, Elastic Peak Electron Spectroscopy (EPES) and its positron counterpart provide valuable insights into the scattering processes of these charged particles in polymeric materials such as polyethylene. The elastic scattering cross section, recoil energy, and inelastic mean free path (IMFP) all play crucial roles in shaping the observed spectra, making a detailed investigation of these effects essential.

It is well-known that in the energy spectra of electrons, a prominent peak, the so-called elastic peak, can be observed, which collects all electrons scattered elastically from solid or liquid targets [1,2,3,4,5,6,7,8,9,10,11,12,13]. Electron Compton scattering (ECS) (also EPES) and positron Compton scattering (PCS) are methods that focus on studying the shape of the elastic peak of electrons and positrons, respectively. Due to the energy transferred to target atoms, the energy of elastically scattered electrons (positrons) is reduced so that the elastic peak is shifted from the beam’s initial kinetic energy [3,5,14].

EPES is a method for the detection and quantification of surface hydrogen [6,8,15]. It has been used in particular to detect hydrogen in polymers such as polymethyl methacrylate [9,10], polystyrene [9,11], and polyethylene [12,16,17]. The reason for the particular effectiveness of EPES in quantitatively determining the presence of hydrogen in polymers lies in the fact that the recoil energy of hydrogen is greater than that of carbon and oxygen. Taking into account the typical instrumental resolution and the initial energy distribution of the incident particles, two well-separated elastic peaks can be observed at a kinetic energy of the incident particles of more than ≈1 keV, one of which is due to hydrogen and the other to the heavier elements that make up the polymer and whose intensities are related to the interplay between elastic and inelastic scattering cross sections.

Please note that polymers are sensitive to irradiation with electron beams, which lead to bond breakage and depletion of hydrogen in the investigated region [9,10,15].

If the targets are insulators and the number of secondary electrons emitted from the surface is less than the number of electrons absorbed in the sample, the surface stabilizes at a potential energy that changes the impact energy and modifies the energy difference between the elastic peaks of hydrogen and carbon. We addressed this issue in our previous work [11,12].

The cited studies showed that EPES can be used for the detection and quantification of hydrogen in polymers by utilizing the distinct recoil energy associated with light elements. However, while the scattering behavior of electrons in polymers has been extensively studied, the comparative study of electron and positron scattering remains less explored. This gap is particularly important as positron interactions provide unique information about material structure and electronic properties.

In this article, we focused on the difference between ECS and PCS spectra of electrons incident on amorphous low-density polyethylene (LDPE) with a density of 0.93 g/cm^3^. We found that in the incident particle energy range from 1 keV to 3 keV, the intensities of the elastic electron–hydrogen peaks are quite similar to those of the elastic positron–hydrogen peaks, with the elastic electron–hydrogen peaks below 1.5 keV being slightly more intense than the elastic positron–hydrogen peaks, which is not the case for the elastic carbon peaks. In fact, the elastic positron–carbon peaks are systematically less intense than the elastic electron–carbon peaks, with the difference in intensity decreasing as the energy of the incident particles increases.

We also calculated the elastic reflection coefficient for the normal incidence on the incident beams. It is defined as the area under the elastic peaks of hydrogen and carbon assuming that the angles accepted by the detector are between 0 and 90 deg with respect to the normal to the surface (scattering angles at normal incidence from 90 to 180 deg) so that the detector captures all electrons (positrons) emerging from the polyethylene surface.

In this work, we perform a systematic comparison of the spectra of elastic electron and positron scattering in polyethylene, focusing on the relative intensities of the elastic peaks and the role of IMFP variations. Using Monte Carlo simulations, we investigate how different IMFP models affect the spectral properties and discuss the implications of our results for experimental studies. By extending previous research and incorporating a wider range of theoretical and experimental comparisons, this study aims to refine existing models and provide new insights into the elastic interactions of electrons and positrons with polymeric materials.

To ensure that this work provides a more in-depth and structured analysis that goes beyond a simple comparison of data, we adopted a methodological approach that considers both the fundamental physical mechanisms underlying elastic scattering and their implications for electron spectroscopy of polymers. The analysis not only compares the intensities of the elastic peaks for electrons and positrons but also investigates the role of elastic scattering cross sections, inelastic mean free paths, and recoil effects. Furthermore, we investigate the effects of different models of the inelastic mean free path on the spectral properties through Monte Carlo simulations to provide a more comprehensive understanding of the phenomenon and its potential experimental applications.

## 2. Theory

### 2.1. Theoretical Framework

If $\langle E_{r}\rangle$ represents the mean recoil energy given by

(1) $$\langle E_{r}\rangle \;=\;\frac{q^{2}}{2M}\;$$

where *q* is the transferred momentum for a target atom with mass *M*, then the recoil energy $E_{r}$ can be calculated by

(2) $$E_{r}\;=\;\langle E_{r}\rangle \;+\;\Delta \;,$$

where Δ is the spread in the recoil energy due to atomic vibrations. This spread can be described by a Gaussian distribution whose standard deviation σ is given by [3,9,18]

(3) $$\sigma \;=\;\sqrt{\frac{4}{3}\langle E_{r}\rangle \langle E_{k}\rangle }\;,$$

where we indicate the mean kinetic energy of the target atoms as $\langle E_{k}\rangle$.

If *E* is the energy of an incident particle (electron or positron) hitting an atom, *m* is the incident particle mass, and θ is the scattering angle, then $\langle E_{r}\rangle$ can be calculated using the equation

(4) $$\langle E_{r}\rangle \;=\;\frac{4m}{M}\;E\;\sin^{2}\frac{\theta }{2}\;.$$

### 2.2. Monte Carlo Method

Let us here describe the most important features of the Monte Carlo method [13,19,20,21].

Let us assume spherical coordinates (r,θ,ϕ). An electron or positron beam irradiates the target surface (which lies in the plane z=0) with the primary energy $E_{0}$ and the angle of incidence $\theta_{0}$.

The elastic scattering cross section $\sigma_{\mathrm{el}}$ is calculated by

(5) $$\sigma_{\mathrm{el}}\;=\;n_{C}\sigma_{C}\;+\;n_{H}\sigma_{H}\;.$$

where $\sigma_{C}$ stands for the elastic scattering cross sections of carbon, $\sigma_{H}$ for the elastic scattering cross sections of hydrogen, and $n_{C}$ and $n_{H}$ for their respective atomic concentrations.

The inelastic scattering cross section $\sigma_{\mathrm{inel}}$ is calculated by

(6) $$\sigma_{\mathrm{inel}}\;=\;\frac{1}{N\lambda_{\mathrm{inel}}}\;.$$

In this equation, $\lambda_{\mathrm{inel}}$ is the inelastic mean free path of the electrons and *N* is the number density. The probabilities for elastic and inelastic scattering are given by

(7) $$p_{\mathrm{el}}\;=\;\frac{\sigma_{\mathrm{el}}}{\sigma_{\mathrm{el}}\;+\;\sigma_{\mathrm{inel}}}$$

and

(8) $$p_{\mathrm{inel}}\;=\;\frac{\sigma_{\mathrm{inel}}}{\sigma_{\mathrm{el}}\;+\;\sigma_{\mathrm{inel}}}\;=\;1\;-\;p_{\mathrm{el}}\;,$$

respectively.

The mean free path is given by

(9) $$\lambda \;=\;\frac{1}{N(\sigma_{\mathrm{el}}\;+\;\sigma_{\mathrm{inel}})}$$

and the step length Δs between the collisions by

(10) $$\Delta s=-\lambda \ln(\mu_{1})$$

where $\mu_{1}$ is a random number that is sampled with a uniform distribution between 0 and 1. Another random number $\mu_{2}$ is sampled with a uniform distribution between 0 and 1 for the choice between elastic or inelastic collisions. If $\mu_{2}\;>\;p_{\mathrm{el}}$, the collision is inelastic. In this case, it is no longer of interest to follow the trajectory of this electron (positron). The simulation of its trajectory is now complete. If, on the other hand, $\mu_{2}\;\leq \;p_{\mathrm{el}}$, then the collision is elastic. In this case, another random number $\mu_{3}$ is sampled uniformly between 0 and 1 to determine the type of elastic collision and the recoil energy. If

(11) $$0\leq \mu_{3}\leq \frac{n_{C}\sigma_{C}}{\sigma_{\mathrm{el}}}\;,$$

then an electron–carbon collision takes place and the scattering angle θ is determined by

(12) $$\mu_{4}\;=\;P_{C}\left(\theta \right)\;,$$

where $P_{C}\left(\theta \right)$ is the cumulative probability of elastic scattering in C and $\mu_{4}$ is a random number sampled with a uniform distribution between 0 and 1. The azimuth angle ϕ is sampled uniformly between 0 and 2π.

The recoil energy is calculated according to Equations (2)–(4), so that

(13) $$E_{r}\;=\;\frac{4m}{M_{C}}E\sin^{2}\frac{\theta }{2}\;+\;\Delta_{C}\;.$$

where $\Delta_{C}$ describes the Doppler broadening in C (Δ in Equation (2)). If

(14) $$\frac{n_{C}\sigma_{C}}{\sigma_{\mathrm{el}}}<\mu_{3}\leq 1\;,$$

then an electron–hydrogen collision takes place and the scattering angle θ is determined by

(15) $$\mu_{4}\;=\;P_{H}\left(\theta \right)\;,$$

where $P_{H}\left(\theta \right)$ is the cumulative probability of elastic scattering in H and $\mu_{4}$ is a random number sampled with a uniform distribution between 0 and 1. Furthermore, in this case, the azimuth angle ϕ is sampled uniformly between 0 and 2π.

According to Equations (2)–(4), the recoil energy is calculated as follows:

(16) $$E_{r}\;=\;\frac{4m}{M_{H}}E\sin^{2}\frac{\theta }{2}\;+\;\Delta_{H}\;.$$

where $\Delta_{H}$ describes the Doppler broadening in H (Δ in Equation (2)) (since $\Delta_{C}$ and $\Delta_{H}$ are determined using random numbers derived from a Gaussian distribution with the standard deviation calculated according to Equation (3), they can be positive or negative).

Please note that $M_{C}$ and $M_{H}$ represent the atomic masses of carbon and hydrogen, respectively, *m* stands for the electron (positron) mass, and *E* is the electron (positron) energy.

The trajectory of each electron (positron) is followed until its energy remains greater than a certain threshold value $E_{t}$, and its coordinate *z* (measured from the surface and directed into the interior of the solid) remains greater than zero.

### 2.3. Cross Sections

**Figure 1:**
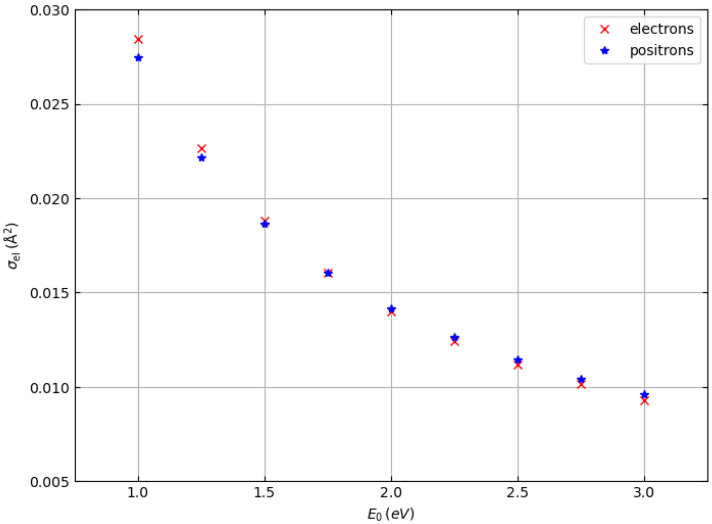
Total elastic scattering cross section of electrons (×) and positrons (∗) hitting hydrogen atoms.

**Figure 2:**
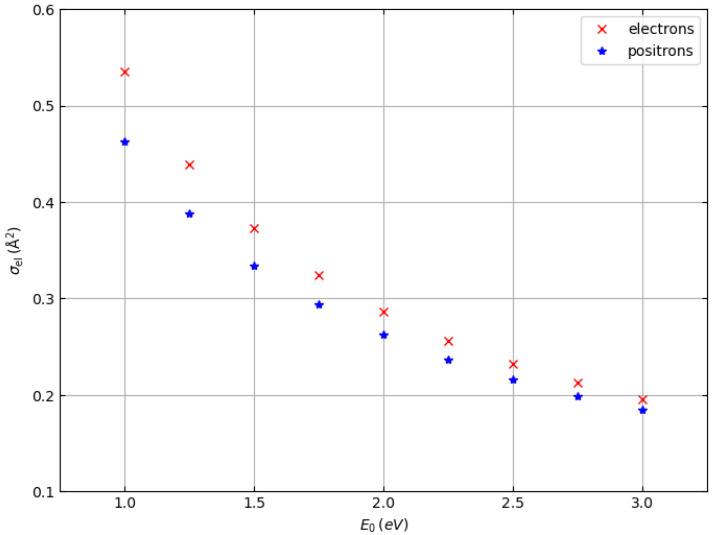
Total elastic scattering cross section of electrons (×) and positrons (∗) hitting carbon atoms.

The total elastic scattering cross sections of electrons and positrons are shown in Figure 1 (target: hydrogen atoms) and Figure 2 (target: carbon atoms) as a function of the incident particle energy in the range from 1.0 to 3.0 keV. They were determined by numerical integration of the Dirac equation in a central field (Mott theory) [20,22]. The Hartree–Fock atomic potential was used and exchange effects were taken into account for the case of electron–atom scattering by using the Furness and McCarthy local approximation [23].

While for hydrogen atoms the total elastic scattering cross sections are practically identical (except at energies of 1 keV and 1.25 keV, where the elastic scattering cross sections of electrons are slightly higher than the elastic scattering cross sections of positrons), for carbon atoms the elastic scattering cross sections of electrons are systematically higher than the elastic scattering cross sections of positrons for all energies studied. Therefore, we expect that we can also observe differences in the shape of the elastic peaks of electrons and positrons of polymers such as polyethylene, which consists of hydrogen and carbon atoms and whose chemical formula is (C_2_H_4_)_n_. In particular, we expect the elastic carbon peak of positrons to be less intense than the elastic carbon peak of electrons, while the elastic hydrogen peaks of positrons and electrons should be almost identical (except for the spectra at about 1–1.5 keV, where the positron peak should be slightly less intense).

Not only the elastic scattering cross section of carbon and hydrogen but also the inelastic mean free path (IMFP) of electrons and positrons in polyethylene plays a role in determining the elastic peak. In fact, the intensity of the elastic peaks in the Compton electron and positron spectra depends on the interplay between elastic and inelastic processes. This is because the inelastic scattering collisions remove intensity from the elastic peak. If you know the density of the target (for low-density polyethylene: 0.93 g/cm^3^ according to ref. [24] (this is the value used in this work) or 0.92 g/cm^3^ according to ref. [25]), you can determine the inelastic scattering cross section from the inelastic mean free path of the electrons and positrons. Using the dielectric theory [26] and optical data [27], the values of the inelastic mean free path in different materials have been calculated by many authors, e.g., by Tung et al. [28] (electrons), by Penn [29,30] (electrons), by Ashley [31] (electrons and positrons), by Tanuma, Powell, and Penn [24,32] (electrons), and by Shinotsuka et al. [25,33]. See Table 1 for the inelastic mean free path in polyethylene in the energy range of interest for the this work. For the comparisons between electrons and positrons presented in this article, we used Ashley’s inelastic mean free paths [31] as they are available for both electrons and positrons. To investigate the effect of the inelastic mean free path model on the elastic peak, we compared the spectra of 2000 eV electrons obtained with the calculations of Tanuma, Powell, and Penn [24] and Ashley [31].

## 3. Results and Discussion

It is known that the elastic peak of electrons impinging on polyethylene has two well-defined features. They are due to the recoil energy, which is higher the lower the atomic mass of the target is, so that the energy shift of electrons elastically scattered on hydrogen atoms is higher than the energy shift of electrons elastically scattered on carbon atoms [15]. Of course, this also applies to elastic positron scattering.

The shape of the elastic peak in electron Compton scattering (and also in positron Compton scattering) depends on many other factors, which we have already discussed elsewhere [10,11,12]. In particular, the Doppler effect, which is related to the mean kinetic energy of the atoms around their equilibrium position, is responsible for the broadening of the peaks [3]. If the incident beam is not perfectly monochromatic, the width of the peaks also depends on the energy distribution of the beam. The instrumental resolution also contributes to the peak width. Other phenomena determine the observed shape. We mention here in particular (i) hydrogen desorption stimulated by particle irradiation and (ii) charge phenomena that occur when the target is an insulator.

Since we are interested in a purely methodological investigation, we intentionally omit discussion of many of these phenomena in this article in order to focus our attention on the differences in the electron and positron spectra.

The spectra presented were obtained by neglecting hydrogen desorption, as this can in principle be reduced by very gentle conditions (low current and short irradiation times).

Charge phenomena are very important, as the incident energy can be altered by the electric field generated by the irradiation at the surface so that the landing energy deviates from the primary energy. This in turn affects the recoil energy and the energy difference between the elastic peaks of hydrogen and carbon. The energy of the incident particles discussed in this paper must thus to be regarded as the landing energy.

Moreover, we consider purely monochromatic beams and neglect the broadening of the peaks due to beam initial energy distribution and analyzer resolution. Therefore, the only broadening source considered here is the broadening resulting from the Doppler effect (Doppler broadening).

All the effects mentioned but ignored (in the current calculations) are of course important to describe a realistic experiment but can lead to confusion when we want to study the differences of electron and positron spectra. Please note that they were all discussed in our previous publications dedicated to the comparison with experimental data to validate the Monte Carlo code [10,11,12].

With this simplified approach, we can isolate the most important phenomena that characterize the spectra, namely, the interplay between the elastic and inelastic mean free paths.

**Figure 3:**
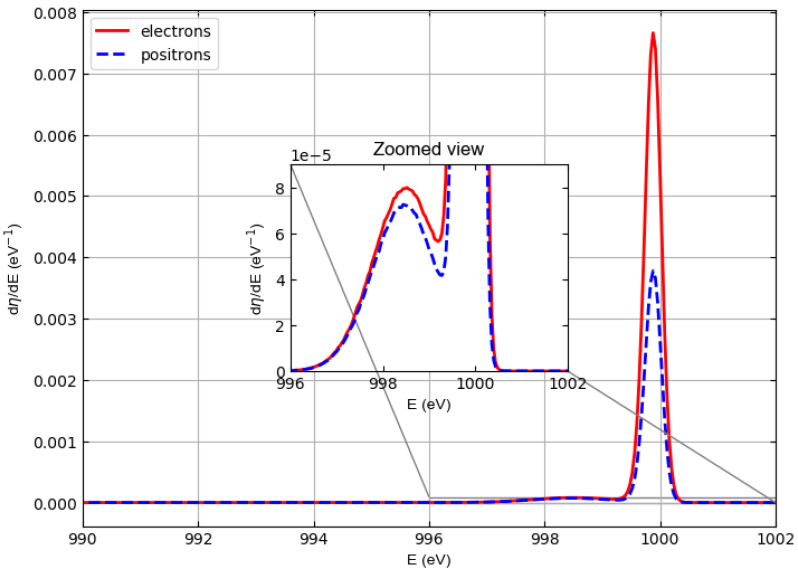
Spectra of electrons (solid line) and positrons (dashed line) elastically backscattered from polyethylene. Incident particle energy $E_{0}\;=\;1000$ eV. It is assumed that the incident beams are monochromatic. With respect to the normal to the surface, the angle of incidence is 0 deg and the angles accepted by the detector are between 0 and 90 deg.

Figure 3 compares the spectra of 1000 eV electrons and positrons elastically backscattered from polyethylene. These spectra were obtained assuming that the incident beams were monochromatic, and, with respect to the normal to the target surface, the angle of incidence was 0 deg and the angles accepted by the detector were between 0 and 90 deg.

The figure shows that the most important elastic peak (which is due to the collision with the carbon atoms) shifts to energies lower than 1000 eV for both electrons and positrons. This shift represents the recoil energy, i.e., the kinetic energy transferred to the target atom during the elastic collision. The enlarged view shows a stronger shift of the elastic peak for hydrogen.

Please note that in the current simulations, as mentioned above, the width peak is only the result of Doppler broadening, which depends on the mean kinetic energy of the target atoms $\langle E_{k}\rangle$ (here taken from experimental data [34]) and on the recoil energy $\langle E_{r}\rangle$ = $4\left(m/M\right)E\sin^{2}\left(\theta /2\right)$, (*m* = incident particle mass, *M* = target atom mass, *E* = incident particle kinetic energy, θ = scattering angle). A Gaussian distribution is observed whose standard deviation σ is given by $\sigma =\sqrt{4\langle E_{r}\rangle \langle E_{k}\rangle /3}$.

**Figure 4:**
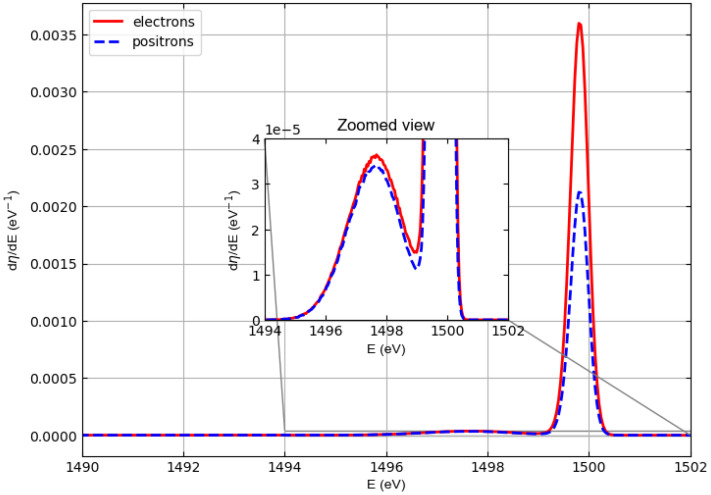
Same as Figure 3 but with incident particle energy $E_{0}\;=\;1500$ eV.

**Figure 5:**
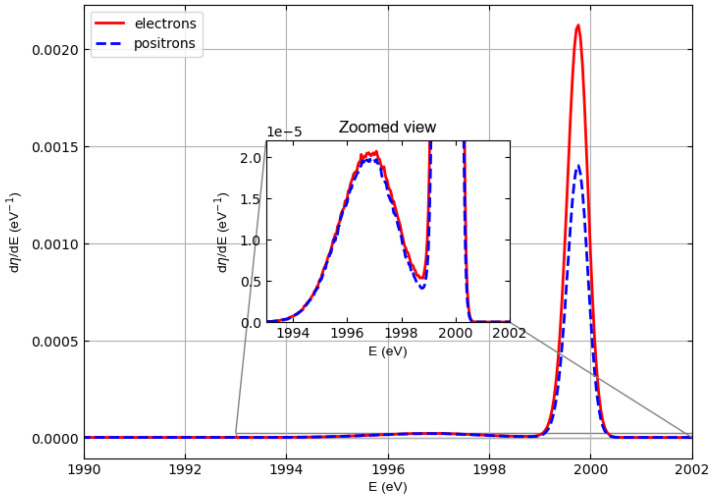
Same as Figure 3 but with incident particle energy $E_{0}\;=\;2000$ eV.

**Figure 6:**
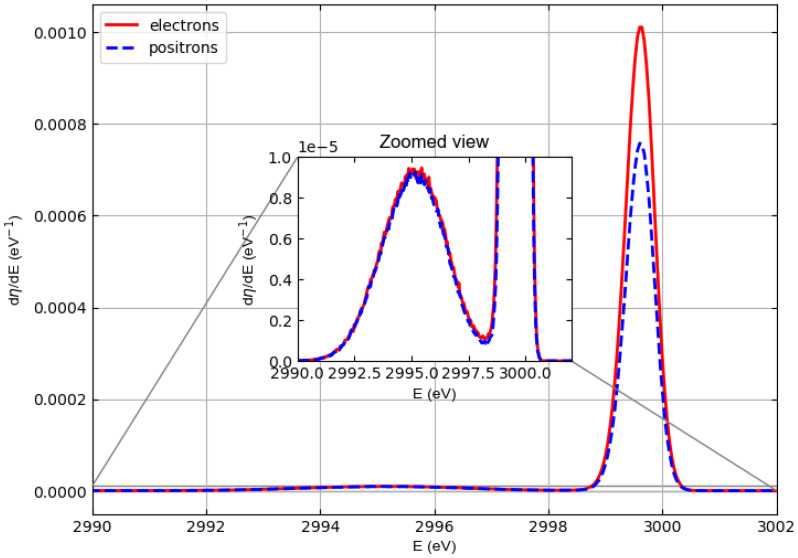
Same as Figure 3 but with incident particle energy $E_{0}\;=\;3000$ eV.

Figure 4, Figure 5, and Figure 6 show similar spectra but with incident particle energies of 1500 eV, 2000 eV, and 3000 eV, respectively.

In general, the cross sections of elastic electron scattering are higher than the cross sections of elastic positron scattering in carbon (see Figure 2). The inelastic mean free paths of electrons and positrons are not very different, so the results represented in the simulated spectra, where we can see that the intensity of the carbon–electron elastic peak is higher than the intensity of the carbon–positron elastic peak, are not surprising.

At an energy of 1000 eV, this can also be observed for hydrogen, although in this case it is difficult to evaluate elastic peak intensities for hydrogen due to the interference with the low-energy tail of the carbon elastic peak. At higher energies (i.e., 1500, 2000, and 3000 eV), the hydrogen and carbon elastic peaks are better separated so that the interference of the high-energy tail of the hydrogen peaks with the low-energy tail of the carbon peaks is less and less relevant. The general behavior is as follows. The intensities of the hydrogen peaks for electrons and positrons are very similar (practically identical at 2000 and 3000 eV), while the difference in the intensities of the carbon peaks decreases with increasing energy, which is consistent with the behavior of the elastic scattering cross sections in Figure 1 and Figure 2.

The elastic reflection coefficient for polyethylene is defined as the area under the hydrogen and carbon elastic peaks assuming that the detector captures all electrons (positrons) emerging from the polyethylene surface. Table 2 shows the elastic reflection coefficient of electrons ($r_{e}$) and positrons ($r_{p}$) defined in this way and multiplied by 100 as a function of the incident particle energy at normal incidence. The data shown in the table were obtained by integrating the spectra in Figure 3, Figure 4, Figure 5 and Figure 6. For both electrons and positrons, the elastic reflection coefficient is a decreasing function of the energy of the incident particles. It should also be noted that although ($r_{p}$) is always smaller than ($r_{e}$), the percentage difference between the elastic reflection coefficient of electrons and positrons decreases as the energy increases.

Table 1 shows that the inelastic mean free path of electrons in polyethylene calculated by Ashley [31] differs from that calculated by Tanuma Powell and Penn [24] (it is higher). This difference obviously also affects the simulation of the spectrum of elastically scattered electrons. In fact, the EPES technique is precisely used to calculate the inelastic mean free path since the inelastic scattering collisions reduce elastic peak intensity.

**Figure 7:**
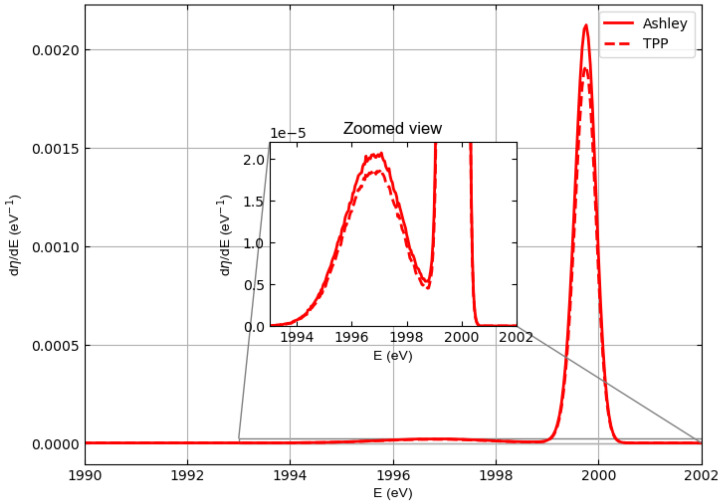
Comparison between the spectra of electrons elastically backscattered from polyethylene obtained using the Ashley IMFP (solid line) [31] and the Tanuma, Powell, and Penn IMFP (dashed line) [24]. Incident particle energy $E_{0}\;=\;2000$ eV. It is assumed that the incident beams are monochromatic. With respect to the normal to the surface, the angle of incidence is 0 deg and the angles accepted by the detector are between 0 and 90 deg.

Figure 7 shows the comparison between the spectra of electrons elastically backscattered from polyethylene obtained with the Tanuma, Powell, and Penn IMFP [24] and the Ashley IMFP [31] for the case where the energy of the incident electrons is $E_{0}\;=\;2000$ eV. Since the Ashley IMFP is higher than the Tanuma, Powell, and Penn IMFP, the Ashley probability of inelastic scattering is lower than the Tanuma, Powell, and Penn probability of inelastic scattering, resulting in a more intense elastic peak. As a result, the electron elastic reflection coefficient for polyethylene multiplied by 100, calculated using the Tanuma, Powell, and Penn IMFP, is smaller than the value given in Table 2 for the same energy and is 0.103 instead of 0.115 (with a difference of the order of 10%).

Not only does the IMFP value used in the simulation influence the spectrum but so, of course, does the acceptance angle. According to Jablonski, for example, the use of the cylindrical mirror analyzer leads to an experimental underestimation of the elastic reflection coefficient [35].

To verify the presented predictions of the elastic reflection coefficient, we calculated it for 2200 eV electrons impinging on amorphous carbon using the IMFP value of 25.2 Å provided by Penn [29]. We found 100×r=0.077. This value agrees quite well with the experimental evidence as it lies between data provided by Jablonski in ref. [35], i.e., the one measured by Gergely (0.062) [36], and the one measured by Schmid et al. (0.080) [37].

## 4. Conclusions

In this study, we used Monte Carlo simulations to systematically compare the elastic scattering interactions of electrons and positrons with polyethylene.

The elastic scattering cross sections for electrons are consistently higher than those for positrons, especially for carbon atoms, and this leads to more intense elastic peaks for electrons. In contrast, the elastic peak intensities for hydrogen are almost identical for both particles, with only slight differences at lower energies.

The choice of IMFP model significantly influences the simulated spectra. For example, the use of Ashley’s IMFP leads to a lower probability of inelastic scattering and thus to a higher intensity of the elastic peaks compared to the results of the Tanuma, Powell, and Penn model.

The simulation results show reasonable agreement with the experimental observations, which confirms both the theoretical framework and the applied methodology. In particular, the elastic reflection coefficients derived from the simulations agree with the available experimental measurements, which underlines the robustness of our approach.

A better understanding of the interactions between charged particles and polymers directly benefits the field of electron spectroscopy and leads to a more precise characterization of materials. The knowledge gained from this study can be used to refine Monte Carlo simulation techniques, which are crucial for predicting electron and positron scattering in various polymer materials. These results have important applications in radiation physics, where accurate modeling of scattering processes is essential.

Further work could extend these investigations to other polymer systems and include additional factors such as hydrogen desorption. Broader experimental validation over a wider energy range and different materials will help to further refine these models and deepen our understanding of elastic and inelastic scattering phenomena.

This work not only reveals the fundamental differences in the scattering behavior of electrons and positrons in polyethylene but also provides a robust framework for improving simulation methods and experimental techniques. These advances pave the way for improved materials analysis and innovative applications in electron spectroscopy and materials science.

For the sake of clarity, we summarize the most important results by single points: (i) The elastic scattering cross sections for electrons are generally higher than those for positrons, with particularly marked effects for carbon atoms. (ii) The difference between the elastic peaks of electrons and positrons is more pronounced at lower energies and tends to decrease as the incident energy increases. (iii) The choice of the model for the inelastic mean free path significantly affects the simulated spectra, as shown by the comparison between the results obtained with the Ashley model [31] and the model of Tanuma, Powell, and Penn [24]. Finally, applications of these results could extend to the characterization of polymeric materials by electron spectroscopy and the development of improved Monte Carlo simulation models for electron–matter interactions.

## Figures and Tables

**Table 1:** Inelastic mean free path of electrons and positrons in polyethylene. The reported values are rounded according to their estimated uncertainty.

Energy (eV)	IMFP	IMFP	IMFP	IMFP
	(Electrons)	(Electrons)	(Electrons)	(Positrons)
	(Å)	(Å)	(Å)	(Å)
	Ref. [25]	Ref. [24]	Ref. [31]	Ref. [31]
1000	-	31.8	35.7	34.8
1096.6	35.2	-	-	
1500	-	44.0	48.9	48.0
1636.0	48.4	-	-	
2000	-	55.6	61.5	60.7
2208.3	61.7	-	-	
3000	-	-	85.6	84.8

**Table 2:** Elastic reflection coefficients of electrons ($r_{e}$) and positrons ($r_{p}$) in polyethylene multiplied by 100 and their percentage differences as a function of the incident particle energy in the range from 1.5 to 3.0 keV at normal incidence. With respect to the normal to the surface, the angle of incidence is 0 deg and the angles accepted by the detector are between 0 and 90 deg so that the detector captures all electrons (positrons) emerging from the polyethylene surface. The reported values are rounded according to their estimated uncertainty.

Incident Particle Energy	$r_{e}\times 100$	$r_{p}\times 100$	$\left(r_{e}-r_{p}\right)/r_{e}$
(keV)			(%)
1.0	0.290	0.147	49
1.5	0.168	0.101	40
2.0	0.115	0.0770	33
3.0	0.0678	0.0514	24

## Data Availability

The original contributions presented in this study are included in the article. Further inquiries can be directed to the corresponding author.
